# Working Women in Large Towns

**Published:** 1890-05-10

**Authors:** 


### Working Women in Large Towns.

### II.—STREET WORKERS.

We propose, in this series of articles, to consider the condition, social and economic, of working women in our large towns, not because we claim to have any new and startling discoveries to make known, or any original remedies to propose, but because we believe the subject to be one which, shamefully neglected in the past, cries out to us to take it up, and, if we cannot go into the matter for ourselves, at least to make ourselves acquainted with the facts which have in various ways been made public. The plea that we are perfectly ignorant of the terrible cost at which some article which we carelessly use is produced will not serve us now. We may not always see our way to remedying the present state of things—that is another matter; but most certainly it will never be remedied by an ignorance which must henceforth be pronounced wilful.

At the same time, we have no wish to go in for sensation, or to speak as if the lot of our working women was one dead level of misery and privation. Many of the work-girls of London would be amazed if you offered them pity, spite of low wages and a low standard of living; while there are plenty of skilled workers of a higher grade who are really arning good wages, and who should be, and doubtless are, living comfortable, respectable, happy lives. But while we must, in mere fairness to ourselves and our civilisation, remember this, the fact remains that this boasted civilisation of ours allows—possibly even causes—a terribly large class of the community to be beaten down, neglected, harried almost out of life itself.

Roughly speaking, the working women whose lot we have to consider may be divided into five classes—Street Workers, Factory and Workroom Hands, Home Workers, Domestic Servants, and Shop Assistants and Barmaids. That there are all sorts of grades and distinctions in these five classes will be evident at once, as is also the fact that the divisions between them cannot in the nature of things be clean-cut, owing to Class III., that of home-workers, being rather supplementary to Class II., than having independent industries of its own. Still, the whole question of home-work is such a difficult and important one, that we have thought well to consider it separately. But let us take the classes in the order in which we have placed them, and begin with the comparatively small one (it is said to contain some two thousand members) which heads our list.

Although there are various trades in which women and girls whose working day is spent in the streets may engage, we shall consider them all together; for, so far from there being any hard and fast line between flower girls, orange and match sellers, and watercress girls, the very same girls will often take up first one of these trades, and then another, according to the time of year. There are long "slack seasons" in flower selling, as in so many other industries, and at these times the poor girls are glad to turn their hands to anything which may enable them to pick up a living. At its best the life of these street sellers is but a hard one. They have to be in Covent Garden by five o'clock in the morning, whatever the weather may be, if they wish to get the pick of the flowers; and when money is scarce with them they must wait about for hours, hoping at last to pick up some cheap blossoms which others have despised. Some are very clever, both at bargaining and at making the very most of their purchases; others are less nimble as regards both wits and fingers, and perhaps sorely hampered by lack of funds; but all will have left the market before nine o'clock, and after making up their flowers into bunches and buttonholes, will wend their way to their accustomed stations in City or West-End—the pavement by the Exchange being considered, we believe, one of the very best of these stations. There they will stand until their wares are sold, in heat or cold, snow or rain; and as they very often have but scanty protection against the inclemency of the weather, we cannot be surprised to hear that it is no infrequent thing for a flower girl to die of consumption.

The mothers of these girls are very generally street sellers themselves, and make use of their children at a very early age, setting them to pick over walnuts for two or three hours before school time, and sending them into the streets at thirteen, or even less, if they can evade the School Board officer. It is a dangerous life for young girls; and if this must be said of flower-selling by day, what of the temptations to which those girls are exposed who go out at night, standing outside the theatres and public-houses, to sell to those who pass in and out, and often staying out till three in the morning? We cannot wonder that flower girls are as a rule of a low class—improvident, dirty, fond of drink, rough in their manners, and loose in their morals. Still, they are by no means without their virtues. They are extremely friendly to each other, and will share their last crust with a "mate" in distress; and the devotion with which many of them will slave for the lazy, good-for-nothing lads whom they marry—or rather, do *not* marry—has something really touching about it. We hear of cases in which a young fellow will live on one of these girls for years, until at last, growing tired of her, he goes off to try his luck elsewhere; and of others in which the poor young breadwinner is so brutally ill-treated by her "husband," that she is forced to fly, and hide herself from him. Still, some of these unions no doubt prove fairly happy ones, the man (who is probably a street seller also) contributing his share to the support of the family. Perhaps the couple may even arrive at that coveted distinction of the street-hawker, the possession of a barrow, from which the man may sell sweets or chestnuts, and the woman serve out little saucers of that dainty of the London streets—whelks. But comparatively few flower girls get such a rise in life as this denotes, and it is to be feared that very many of them undergo rapid demoralisation in their street-life, and "sink to rise no more."

We have spoken above of the improvidence of these girls, of which the principal cause is, no doubt, the extreme uncertainty of their trade. They literally live from hand to mouth, now feasting for a few days, now all but starving. Still, the fact that many of them form Feather Clubs among themselves shows that they are not utterly heedless of the advantages to be gained by "putting by;" while when money is comparatively plentiful, they will often buy a gold ring, or a handsome shawl, thus guarding themselves from frittering it away, by exchanging it for a safer kind of "portable property," upon which money can yet be raised in times of exceptional need. Another hopeful sign is that such of them as apply for grants from the Emily Loan Fund (instituted by Lord Shaftesbury), to enable them to purchase their stock-in-trade, pay back these loans most faithfully.

We may briefly mention here the work of the Clerkenwell Watercress and Flower Girls' Mission, of which this loan fund is a branch. The workers here try by every means in their power to raise, civilise, and help the girls engaged in this perilous trade; besides which, they endeavour to wean from it children who have no guardians, or worse than none, training them for service, or teaching them artificial flower making. In a bright, cheerful room at the mission quarters in Clerkenwell Close may be seen any day some twenty to thirty girls, busily engaged with irons, wire, and paste in various processes, from which finally emerge red and white roses, bunches of daisies, and long trailing sprays of golden buttercups and other flowers, such as would do credit to really high-class shops. No doubt the bright masses of colour please the eyes of the little workers, accustomed as they have always been to rest on pretty things; and as over 700 young flower-sellers have been induced to exchange their trade for this, or for service, it is evident that this branch of the mission has not been unsuccessful. At the same time we feel that the most important part of its work is that which helps the flower sellers in and not out of their present life. The selling of flowers in the streets is likely to increase rather than diminish; and if we could persuade all the flower-sellers in London to give up their trade, others would at once flock into it. In the main, then, our efforts can only be usefully directed to providing what safeguards we can for these street workers, and bringing influences to bear upon them which may help them to resist temptation, whether it be to reckless improvidence, or to sin.

Since the above lines were written, the "London Flower Girls' Guild" has been inaugurated—an institution which is intended for the benefit of West-London flower girls, and to which we cordially wish success.

